# Semiautomated Multimodal Breast Image Registration

**DOI:** 10.1155/2012/890830

**Published:** 2012-02-01

**Authors:** Charlotte Curtis, Richard Frayne, Elise Fear

**Affiliations:** ^1^Department of Electrical and Computer Engineering, University of Calgary, Calgary, AB, Canada T2N 1N4; ^2^Department of Radiology, University of Calgary, Calgary, AB, Canada T2N 1N4

## Abstract

Consideration of information from multiple modalities has been shown to have increased diagnostic power in breast imaging. As a result, new techniques such as microwave imaging continue to be developed. Interpreting these novel image modalities is a challenge, requiring comparison to established techniques such as the gold standard X-ray mammography. However, due to the highly deformable nature of breast tissues, comparison of 3D and 2D modalities is a challenge. To enable this comparison, a registration technique was developed to map features from 2D mammograms to locations in the 3D image space. This technique was developed and tested using magnetic resonance (MR) images as a reference 3D modality, as MR breast imaging is an established technique in clinical practice. The algorithm was validated using a numerical phantom then successfully tested on twenty-four image pairs. Dice's coefficient was used to measure the external goodness of fit, resulting in an excellent overall average of 0.94. Internal agreement was evaluated by examining internal features in consultation with a radiologist, and subjective assessment concludes that reasonable alignment was achieved.

## 1. Introduction

2D X-ray mammography is the current gold standard breast cancer screening and diagnostic imaging modality [1]. However, mammography has been shown to have low sensitivity and specificity among premenopausal women and women with dense breasts [2]. Furthermore, mammography provides limited 3D information, as only two images are obtained: one in the cranial-caudal (CC) and one in the medial-lateral oblique (MLO) direction. Finally, the breast is compressed up to 50% of its original diameter, resulting in an image with significant anatomical distortion [3].

To overcome these limitations of mammography, other modalities such as magnetic resonance (MR) and ultrasound imaging are used to assist in the diagnosis of symptomatic and high-risk patients. It has been shown that consideration of information from multiple modalities can provide diagnostic information that might be missed if only a single modality was used [2]. As a result, development of novel imaging modalities is an active area of research, as each new technique has the potential to improve diagnosis and ultimately patient outcome.

Tissue sensing adaptive radar (TSAR) is an emerging microwave-based 3D breast imaging modality [4]. Currently, in the early stages of clinical trials, TSAR shows potential as a safe and inexpensive means of obtaining 3D images of the breast. However, as a new type of image, interpretation requires direct comparison with an established modality that provides reference data about the location of internal structures. While current studies use MR images for this purpose, MR images may not be available in future studies involving larger patient cohorts. Fortunately, information about the breast exists in the form of X-ray mammograms, as current clinical protocols specify acquisition of mammograms in almost all cases [1]. These images are a valuable source of information about the internal and external structure of the breast and could potentially be used in place of MR images to assist with TSAR image interpretation.

In order to use mammographic data to assist with TSAR image interpretation, the mammograms must be mapped into the 3D space of the TSAR system. As the first stage of this procedure, this paper focuses on registration of mammograms to 3D image projections in order to obtain an estimate of mammographic features in an undistorted 2D projection space, with the future goal of estimating the location of mammographic features in 3D space. MR images were used in place of TSAR images to develop and test the algorithm, as MR images are an established modality and can be used to assess the effectiveness of the technique. However, it is anticipated that the algorithm will be equally effective on TSAR data when full 3D images become available.

Image registration is a technique used to combine information from multiple images by mapping the coordinates of one image into the space of another. Rigid registration methods, involving only affine coordinate transformations, are both the simplest and most rigorously validated [5]. These techniques are well established in research and are becoming increasingly common in clinical settings, particularly in neuroimaging [5]. Nonrigid registration, requiring nonlinear coordinate transformations, is facing larger resistance to clinical adoption due to issues such as difficulties in validating results as well as increased computational complexity [5].

Breast images present a unique challenge to image registration due to the large and anisotropic deformations resulting from compression during mammography and gravitational forces during MR acquisition [6]. Furthermore, the two modalities differ in dimension, resolution, dynamic range, and intensity/tissue relationship. As a result, breast image registration methods are in their infancy compared to rigid registration application areas.

So far, four approaches to problem of registering mammograms to 3D images have been published. In 2003, Behrenbruch et al. used a landmark-based registration algorithm to align mammograms to “simulated mammograms” created from MR images [7]. Martí et al. took a similar approach in 2004, with the addition of an intensity-based registration technique to determine the appropriate angle for MLO projection of the MR data [8]. Both groups aimed to reverse the effects of mammographic distortion using 2D nonrigid registration techniques.

These registration techniques share similar limitations. As breasts are nonuniform structures, the presence of specific internal landmarks is not guaranteed, which could lead to noncorresponding points being used as alignment points. Furthermore, pathological regions were used as strong landmarks for alignment [7, 8]. However, it is often desirable to compare regions that do not obviously correspond such as microcalcifications seen on mammograms or regions of increased contrast agent uptake on MR images; the dependence on the use of lesions as registration landmarks could inhibit such comparisons.

A drawback of the two aforementioned studies was the prevalence of film mammograms at the time. While film mammograms have high diagnostic quality, such images do not contain information about the MLO acquisition angle and the amount of mammographic compression. As a result, estimation of these geometric factors posed further challenges for researchers during the analog-to-digital transition period [7, 8].

Ruiter et al. took the approach of creating a patient-specific finite element (FE) model based on an 3D MR image, deforming it computationally to simulate mammographic compression, then creating a simulated mammogram through this compressed volume [9]. Following model creation, Ruiter's group achieved correspondence by creating a projection image, updating the boundary conditions of the model by comparing to the original mammogram, then reprojecting through the MR volume. In this manner, compensation of the full 3D effects of mammographic distortion was achieved.

While the results from Ruiter's FE-based method were the most accurate of the three studies, the authors provide the caveat that the method was limited to mammograms acquired under compression of only 21% strain, which is at the low end of the clinical mammographic range [9]. Furthermore, development of a patient-specific FE model is not a trivial task, and as such poses a barrier to clinical use of this technique. Finally, the technique was only applicable to mapping MR images into mammographic space, whereas the current work aims to achieve the opposite in order to assist with TSAR image interpretation without modifying the TSAR data.

Most recently, Mertzanidou et al. deformed a breast tissue model formed from MR data using a 3D affine transformation then created a projection image to compare to a corresponding mammogram [10]. While this method models deformation in 3D, a simple affine transformation is unlikely to capture the highly nonlinear deformation of breast tissue. The authors do not provide quantitative measures of registration accuracy between MR and real mammogram data, so the technique cannot be compared to previous work. Like Ruiter's method, this technique is not suitable for mapping from the mammogram to the space of the MR.

**Figure 1:**
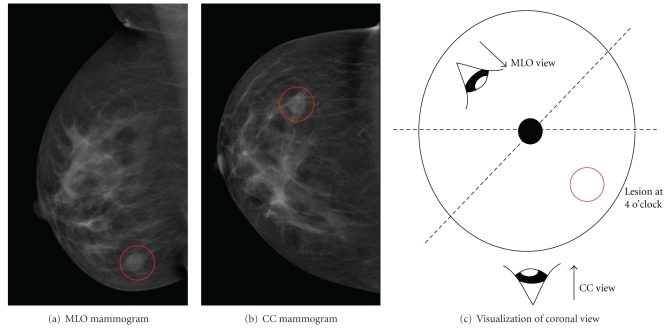
O'clock position of a lesion on a right breast as identified by radiology report.

The algorithm presented in this paper is aimed at obtaining an approximate 2D registration in order to map features seen on mammograms to regions of interest on a projection image formed from corresponding MR data. Due to the levels of deformation involved in acquiring the images, precise pinpointing of the lesion location is not a reasonable expectation. For the purposes of assisting TSAR researchers, the same “hour” of the breast (within 30° of a radial line drawn from the nipple to the lesion in a coronal view) is sufficient. A depiction of a mammogram with a radiologist-identified lesion at 4 o'clock is shown in Figure 1.

Section 2.1 of this paper describes a series of preprocessing steps that are performed on both images to detect landmarks for preliminary gross feature alignment. The mammogram is then mapped into the space of the MR projection image (MRPI) using a two-stage registration method described in Section 2.2.  Section 3 presents validation for this technique using numerical phantom data. Finally, Section 4 shows the results from 24 image pairs from six subjects, quantified using Dice's coefficient and joint entropy plots. Successful registration is demonstrated by both objective and subjective evaluation in all cases.

## 2. Methods

**Figure 2:**
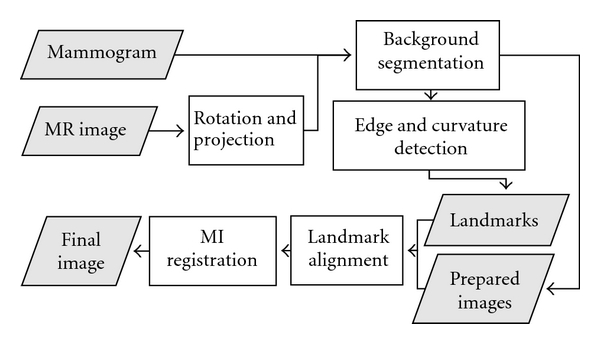
Overview of complete algorithm. Shaded parallelograms represent input/output objects, while rectangles represent processing steps.

Prior to undergoing registration, both image types were preprocessed to remove background noise and identify external anatomical landmarks. These landmarks were used to approximately align the gross features of the images, providing a starting point for precise intensity-based registration. A flow chart illustrating the overall algorithm is shown in Figure 2.

### 2.1. Preprocessing

As coordinate mapping from the mammogram space to the MR image space was desired, the first step in image preprocessing was to create 2D images from the MR volume. While previous work employed models of X-ray attenuation to achieve images in the same intensity range as mammograms, a simple mean intensity projection was chosen for this work as the registration algorithm described in Section 2.2 does not require corresponding intensity values.

**Figure 3:**
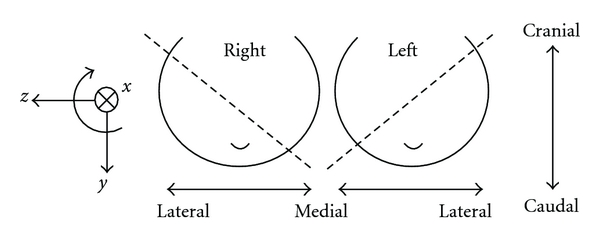
Anatomical directions and cartesian coordinate system. Dotted lines represent MLO image planes.

In digital mammography, the angle of the X-ray beam vector relative to vertical is known and provides an estimate of the acquisition angle of the imaging plane. While this angle may not be exact due to variations in patient positioning, it is likely to be a more precise estimate than assuming a 45° separation between views. Mean intensity projection images were created along each of the CC and MLO X-ray vectors, resulting in magnetic resonance projection images (MRPIs) or “simulated mammograms” of the uncompressed breast.  Figure 3 depicts the coordinate system used in this work in relation to the anatomical directions of the body.

To prepare the mammogram and MRPI for the registration stage, a preprocessing algorithm was used to automatically detect landmarks, remove background noise, and crop away the chest wall; each mammogram and MRPI was processed separately for a total of four images for one breast. Behrenbruch et al. showed that three anatomical points of reference (landmarks) could be used for preliminary image alignment: the nipple and the points of maximum curvature where the breast meets the chest wall, referred to as “rib” and “axilla” [7]. These same landmarks were used in the current work.

In order to segment the breast and background regions, fuzzy connected region growing was used [11]. A seed pixel is chosen as the first member of the segmented region; in this work, the top-left corner pixel was chosen. Each of the neighbours of this pixel is examined, and a connectivity value is computed, and if any of these pixels is determined to be connected, their neighbours are in turn examined. This process continues until all the pixels in the image have been visited [11]. This algorithm produced a new image composed of the fuzzy connectedness values for each pixel relative to the seed pixel, ranging from 0.0 to 1.0. The image was then divided into background and breast regions by thresholding at a fuzzy membership value of 0.5, with an intensity value of 0 assigned to the background region and 1 assigned to the breast region.

The resulting binary segmentation was smoothed using a binary median filter to remove small islands in the segmentation regions and reduce irregularities in the region contours. The line defining the skin/air interface was created by iterating through all the pixels of the foreground (breast) region and examining its 8 connected neighbours. If there was at least one of each background and foreground neighbours, the centre pixel was assumed to be part of the boundary. Finally, every third point on this edge was used as a knot in a cardinal spline to further smooth the contour and simplify curvature computation.

The three anatomical landmarks as defined by Behrenbruch et al. were computed as the points of maximum curvature in the expected portion of the spline bounding box: the left-most 20% for the nipple point and the top and bottom 30% for the axilla and rib points, respectively [7]. These landmarks provide approximate locations of anatomical features to be used for preliminary alignment only; the final registration does not depend on the accuracy of these landmarks.

The curvature *C*(*u*), where *u* is a parameter ranging from 0 to 1.0 along the path of the spline, was computed as follows:

(1) $$C\left(u\right)=\frac{d^{2}u/dx^{2}\cdot du/dy-du/dx\cdot d^{2}u/dy^{2}}{{\left(du/dx\right)}^{2}+{\left(du/dy\right)}^{2}}$$

with derivatives estimated as finite differences.

**Figure 4:**
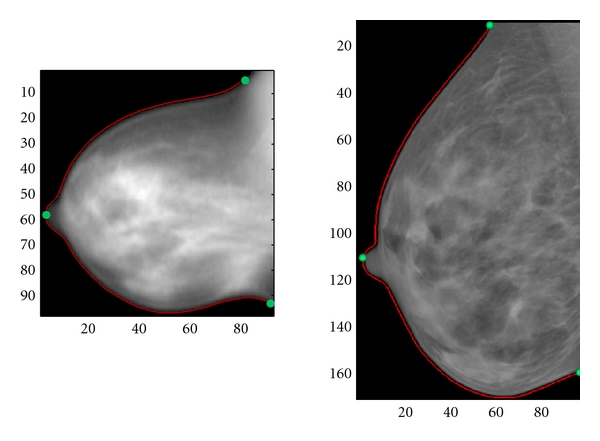
Preprocessed images, contour splines, and landmarks. Due to lateral expansion of tissues during compression, the mammogram (right) is approximately 30% larger than the MRPI (left).

After calculating curvatures, the original mammogram or MRPI was multiplied pixelwise with its binary segmentation image, resulting in a homogeneous zero-intensity background. The image was also cropped around the spline bounding box with a 5-pixel margin to remove the chest wall, ensuring that only tissues in the breast region could influence the registration. Examples of resulting images are shown in Figure 4.

### 2.2. Registration

As evidenced by the images of Figure 4, the external shape of the mammograms and MRPIs is quite different. Most significantly is the difference in surface area; though the two images of Figure 4 are to scale, the mammogram appears significantly larger due to tissue expansion. Preliminary alignment is performed to account for the bulk deformation of the mammogram and provide a reasonable starting point for registration.

To align the gross features of the two images, landmark-based registration was performed using an elastic body spline (EBS) coordinate transform. This technique uses the displacement between corresponding landmarks as control points, and the rest of the pixel locations are interpolated using a spline modelling the physical properties of an elastic body [12].

**Figure 5:**
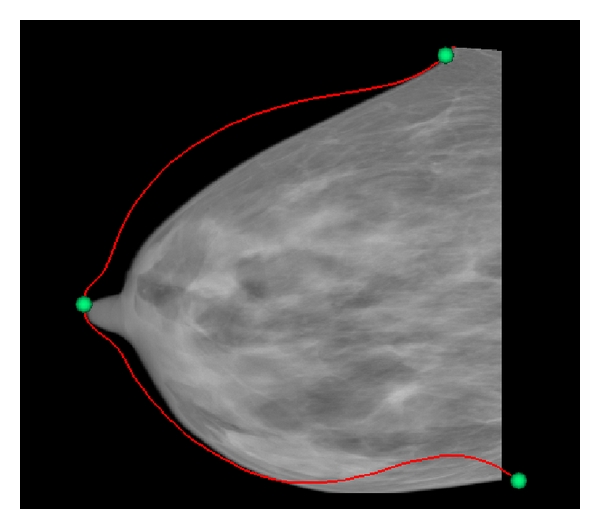
The mammogram of Figure 4 following preliminary alignment with contour and landmarks from the MRPI overlaid.

Using the EBS method, the displacement of a pixel location $\vec{x}=[x,y]$ can be calculated as follows:

(2) $$\vec{d}\left(\vec{x}\right)=\overset{2}{\underset{i=0}{\sum }}G\left(\vec{x}-{\vec{p}}_{i}\right){\vec{c}}_{i}+A\vec{x}+\vec{b},$$

where $G(\vec{x})$ is a 2 × 2 matrix accounting for material elasticity and applied forces, ${\vec{p}}_{i}$ is the coordinates of the 3 landmarks, ${\vec{c}}_{i}$ is spline coefficients, and $A\vec{x}+\vec{b}$ is an affine transform accounting for the bulk displacement, rotation, and scaling of the image. An example of the resulting image is shown in Figure 5.

After obtaining an estimate of the overall deformation of the breast, precise registration accounting for internal tissue distortion can be performed. This deformation was modelled by iteratively computing displacement vectors for a sparse grid of *M* control points ${\vec{\lambda }}_{j}$ with pixel values interpolated on a B-spline basis [13].

Each of the control points ${\vec{\lambda }}_{j}$ has an associated deformation coefficient ${\vec{\delta }}_{j}$ describing the deformation in each of the component directions. The deformation at any image point $\vec{x}$ can be interpolated via

(3) $$D\left(\vec{x}\;|\;\vec{\delta }\right)=\overset{M-1}{\underset{j=0}{\sum }}{\vec{\delta }}_{j}\beta^{\left(3\right)}\left(\frac{\vec{x}-{\vec{\lambda }}_{j}}{\Delta \vec{\rho }}\right),$$

where *β* ^(3)^ is the separable cubic B-spline convolution kernel, ${\vec{\lambda }}_{j}$ is the *M* control points or grid intersection points, and $\Delta \vec{\rho }$ is the grid spacing. In this work, a 6 × 6 grid of control points was typically used.

The transformation of the mammogram is achieved by combining the deformation term of (3) with a bulk rotation **R** and translation **T**:

(4) $$g\left(\vec{x}\;|\;\mu \right)=R\left(\vec{x}-{\vec{x}}_{C}\right)-\left(T-{\vec{x}}_{C}\right)+D\left(\vec{x}\;|\;\vec{\delta }\right),$$

where ${\vec{x}}_{C}$ is the centre of the mammogram. The full set of transformation parameters is given as $\mu =\{\alpha ,t_{x},t_{y};{\vec{\delta }}_{j}\}$, where *α* is the Euler angle of the rotation matrix **R**, *t*_*x*_ and *t*_*y*_ define the translation vector **T**, and ${\vec{\delta }}_{j}$ is the deformation coefficients of (3).

The transformation parameters *μ* were optimized iteratively using a limited memory Broyden Fletcher Goldfarb Shanno optimizer (L-BFGS) [14]. At each iteration, the deformed mammogram was produced and compared to the unchanging MRPI using mutual information (MI) as the optimization metric.

MI is a statistical measure of data similarity, describing how much information can be obtained from one data set given knowledge of another [13]. MI is ideal for intermodality registration, as it does not require corresponding intensity values in the two images; rather, it assumes that regions of homogenous intensity will map into other regions of homogeneous intensity [13]. With the breast images used in this work, this condition is a reasonable assumption, as tissue intensities are consistently different within the two images; for example, in both modalities, fatty tissues are distinct from fibroglandular, though the absolute intensity values do not correspond between the two images.

The MI implementation developed by Mattes et al. was used, as it is more computationally efficient than previous methods [13]. MI was estimated from the image histograms as [13]:

(5) $$S\left(\mu \right)=\overset{N-1}{\underset{\kappa =0}{\sum }}\;\overset{N-1}{\underset{\iota =0}{\sum }}p\left(\kappa ,\iota \;|\;\mu \right)\log_{2}\frac{p\left(\kappa ,\iota \;|\;\mu \right)}{p_{Ma}\left(\iota \;|\;\mu \right)p_{MR}\left(\kappa \right)},$$

where *p*, *p*_*Ma*_, and *p*_*MR*_ are the joint, mammogram marginal, and MRPI marginal probability distributions, respectively, *ι* and *κ* are the integer indices of *N* histogram bins for each image, and *μ* is the set of transformation parameters as in (4). A default of 128 equally spaced bins was used for both data sets, resulting in wider bins for the mammograms.

Using the L-BFGS optimizer, MI was maximized by minimizing the negative value of (5). Optimization was set to stop when the difference between subsequent metric values was less than 10 times machine epsilon (double precision, *ϵ* = 2^−53^) or when a maximum number of iterations was reached. The final transform parameters *μ* were then applied to the mammogram using (4) to obtain the registered image.

Since the MI computation is independent of image resolution, the final registered image maintains the high resolution of mammography while being mapped into the physical space of the lower-resolution MRPI.

All of the algorithms described above were implemented in C++ using the Insight Toolkit (http://www.itk.org) for image processing, the Visualization Toolkit (http://www.vtk.org) for display of images and annotations, and wxWidgets (http://www.wxwidgets.org) for a graphical interface for parameter manipulation.

Both preprocessing and registration algorithms were automated once a set of parameters was determined. These parameters included the number of iterations allowed for registration, the number of histogram bins used to compute MI, the number of knots used in the edge spline, and other factors that required modification on a case-by-case basis. A default parameter set was used to achieve registration in approximately 50% of cases; hence, this algorithm cannot be called fully automated. However, expert intervention (e.g., manual feature identification) was not required to achieve registration.

### 2.3. Evaluation of Results

Registration accuracy is difficult to assess, particularly in the breast where internal landmarks are not readily identifiable or consistent. Furthermore, in the case of images collected with different modalities, arithmetic operations such as subtraction do not provide a meaningful image. Therefore, alternative metrics are required.

For the external breast shape, accuracy was evaluated using Dice's coefficient, computed as

(6) $$D=\frac{2\left|X\cap Y\right|}{\left|X\right|+\left|Y\right|},$$

where |*X*| and |*Y*| are the sizes in physical units of the MRPI and mammogram breast areas (nonbackground pixels) and |*X*∩*Y*| is the size of the overlapping region. Dice's coefficient ranges from 0 (no overlap) to 1 (completely aligned).

Without corresponding internal landmarks, assessment of internal accuracy is not possible. However, pre- and postregistration joint entropy plots of the two images can be examined to visualize the effects of MI maximization. While the entropy plots do not constitute validation of internal feature registration, they do provide confidence that the MI converged to a reasonable maximum.

The MI function *S*(*μ*) of (5) can also be expressed in terms of image entropy or randomness as follows [15]:

(7) S(μ)=E(f)+E(m ∣ μ)−E(f,m ∣ μ),

where *E*(*f*) and *E*(*m*) are the entropies of the fixed (MRPI) and moving (mammogram) images, respectively, and *E*(*f*, *m*) is the joint entropy of the two images; thus, maximization of MI is the same as minimization of joint entropy. The entropy function is defined in terms of the probability distribution as [15]:

(8) $$E\left(x\right)=\overset{X}{\underset{i}{\sum }}p_{X}\left(x\right)\text{lo}{\text{g}}_{\text{2}}\left(p_{X}\left(x\right)\right),$$

where *x* is the pixels in image *X*. The joint entropy is calculated similarly, substituting the joint probability distribution for the marginal distribution *p*_*X*_.

A graphical representation of the joint entropy can be plotted using the image histograms in lieu of the estimated probability distributions; this plot is directly related to the joint image histogram. The joint entropy plot is an *N* × *N* image, where *N* is the number of bins in each image histogram. The intensity value *f* at each pixel location (*i*, *j*) in the joint entropy image is computed as

(9) $$f_{ij}=-p_{ij}\;\log_{2}\left(p_{ij}\right),$$

where *p*_*ij*_ is calculated from the frequency count of the bins of the joint histogram between the two images:

(10) $$p_{ij}=\frac{q_{ij}}{\sum_{i=0}^{N-1}\sum_{j=0}^{N-1}q_{ij}}.$$

The value *q*_*ij*_ is the frequency of the bin *ij* in the joint histogram, calculated as the number of pixels where the fixed image has intensities falling into bin *i* and the moving image has intensities in bin *j* at corresponding locations.

The resulting joint entropy plot is a visualization of pixel correspondence between images, with the scalar MI value representing the amount of dispersion in the joint entropy plot or 2D histogram [15]. Maximizing MI correlates to minimizing dispersion; thus, a joint entropy plot with more coherence has higher MI, which in turn suggests that geometric alignment has been achieved [15].

## 3. Validation Using Numerical Phantom

As with assessment of registration accuracy, validation of registration methods is a difficult task as a ground truth is generally not available [6]. Typical methods of quantifying registration accuracy involve placing fiducials in the object of interest, performing phantom studies, or using clearly defined landmarks within the object [6].

In this work, fiducial placement was infeasible as data were acquired retrospectively. Similarly, no clearly defined landmarks could be identified within the breast. Therefore, a numerical phantom was created and deformed using a corresponding coupled Eulerian-Lagrangian finite element (FE) model [16]. While this model was developed for other purposes, it was sufficiently adaptable to allow for simulation of CC mammographic compression and gravitational deformation.

The FE model was defined as a hemispherical Lagrangian skin surface filled by an Eulerian fluid representative of fatty tissue as well as a sphere of denser Eulerian material to act as glandular tissue [16]. The flat surface of the hemisphere was defined as the chest wall and was not allowed to deform, while a parallel-plate displacement boundary condition was used to mimic mammographic compression in the CC view. A constant force simulating gravity was used for the MR distortion case [16].

**Figure 6:**
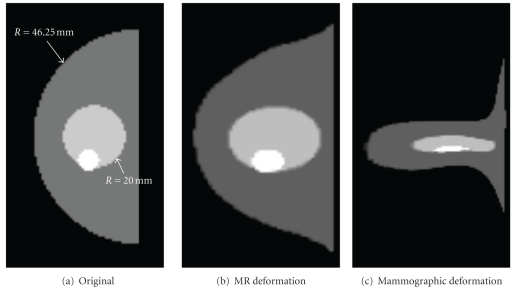
Slices taken from the centre of the numerical phantom following deformation via finite element modelling.

A numerical phantom matching the physical dimensions of the FE model was created with a voxel intensity value of 200 for fatty tissue. Glandular tissue was assigned a voxel intensity of 400, and two smaller spheres with intensity values of 800 were created to represent lesions. While these lesions were not present in the FE model, it is assumed that they do not significantly affect breast deformation. The dimensions of this model are indicated in Figure 6(a).

The point clouds defining the positions of the skin elements of the FE model before and after mechanical deformation were used to deform the numerical phantom using a method similar to the EBS method described in Section 2.2. This procedure was repeated for both CC mammographic compression and MR gravitational distortion. Cross-sections of the original and deformed images are shown in Figure 6.

**Figure 7:**
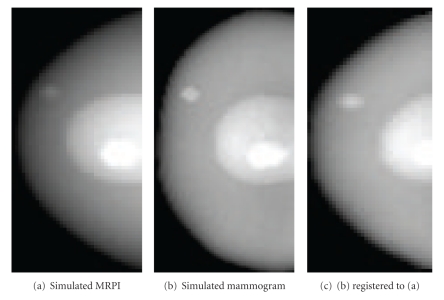
Mean intensity projections through deformed phantom before and after registration.

Average intensity projections through each of the deformed phantoms were taken, resulting in the simulated MRPI and mammogram shown in Figures 7(a) and 7(b). While these images differ in appearance from true clinical images, they are sufficiently different from each other to pose a challenge for registration. In this sense, they should not be considered an attempt to emulate clinical images; they are simply projections of a known 3D geometry following two types of distortion.

The final simulated mammogram following image registration is shown in Figure 7(c). Visual examination of Figures 7(a) and 7(c) indicate that the external shape of the registered simulated mammogram matches that of the simulated MRPI to a high degree of accuracy; this is confirmed with a computed Dice's coefficient value of 0.96. Registration of the large internal structure representing glandular tissue is less successful, as the structure in Figure 7(c) is distinctly different compared to that of Figure 7(a). However, both of the internal lesions match quite closely, with centroids no further than 2 mm (2D Euclidean distance) on the registered images. This falls well within the targeted “same hour” accuracy.

**Figure 8:**
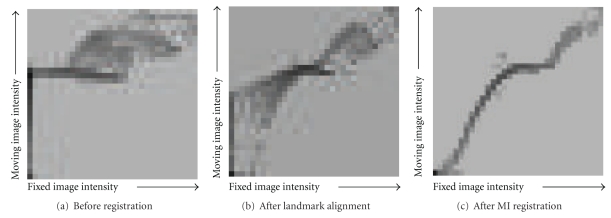
Joint entropy plots of simulated images showing increasing coherence with each registration stage.

Successful registration can also be observed by comparing the joint entropy plots of Figure 8, where the plot prior to registration shows significant disorder and the plot following registration gains cohesiveness. While the original phantom was composed of only three intensity values, the projection images are more complex due to averaging. As a result, the joint entropy plots (shown in Figure 8) are larger than 3 × 3, though still more coherent and simpler than patient data. Figure 8 also shows the value of the MI registration step: though the landmark alignment stage can appear to bring the two images into reasonable alignment (as illustrated with patient data in Figure 5), the internal structures of the breast do not correspond without further registration.

**Figure 9:**
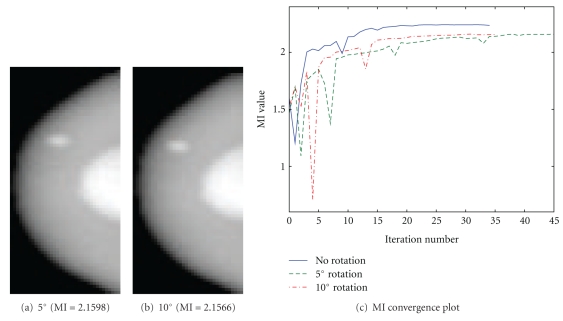
Results of registering simulated images following small misrotations of MR volume. Final images and the trend of MI values over time are shown.

A secondary role of the numerical phantom was to test the sensitivity of the registration algorithm to errors in recorded projection angle resulting from variations in patient positioning. To simulate this, the phantom was rotated around the *x* axis from 0 to 15° in 5° intervals prior to MRPI formation. The resulting registered images, using the parameters determined to be optimal for the zero rotation case, are shown in Figure 9 for the 5- and 10-degree rotations, while the 15-degree rotation case failed to converge.

Figure 9(c) shows the trend of MI values during the iterative registration process for each rotation case. It can be seen that all three converge to a similar MI value, indicating that registration accuracy is not significantly affected by rotation errors of up to 10 degrees. However, the jagged appearance of the 5° and 10° curves of Figure 9(c) indicates instabilities in the optimization, suggesting that errors in projection angle could result in convergence on an incorrect solution or failure to converge altogether, as in the 15° case.

## 4. Experimental Results

The registration process was tested on a total of 24 pairs of images from 6 patients, two with confirmed malignancies. Images were acquired retrospectively and included clinical mammograms and T1-weighted fat-suppressed MR images from a variety of imaging clinics in Alberta, Canada. Gadolinium contrast-enhanced images were also collected, but only the precontrast images were used for registration as they contain more structural information. Data from 10 patients were originally collected, but 4 whose breasts contacted large regions of the MR coil were excluded, as the resulting images were significantly deformed and could not be used as reference images. Moderate deformation was acceptable provided the nipple was not in contact with the bottom of the coil.

Four cases from the patient data sets were selected for further discussion. Where specific image pairs are indicated, they are referred to by the code <subject number>-<breast laterality>-<mammographic view>. For example, the sample data set of Figures 4, 5, and 12 is labelled 091208-R-MLO.

Due to the variety of imaging technicians and machines used, the images varied in acquisition parameters and quality. Mammogram pixels ranged from 0.094 to 0.07 mm square, while MR voxels measured 0.43–0.39 mm in the sagittal plane with 1.12 mm spacing. Despite this variation in imaging parameters and resolutions, the registration technique was successful in all cases, demonstrating a degree of robustness.

With the assistance of a radiologist, pathological regions were identified. Only one data set contained a lesion visible on both modalities, data set 100704-R. The mammograms and location of the lesion as indicated by the radiology report are shown in Figure 1. The lesion was not visible in MRPIs created from the precontrast MR image; however, it is obvious in MRPIs created from the subtraction (postcontrast—precontrast image). These images were compared with the registered mammograms to obtain an estimate of internal feature alignment.

**Figure 10:**
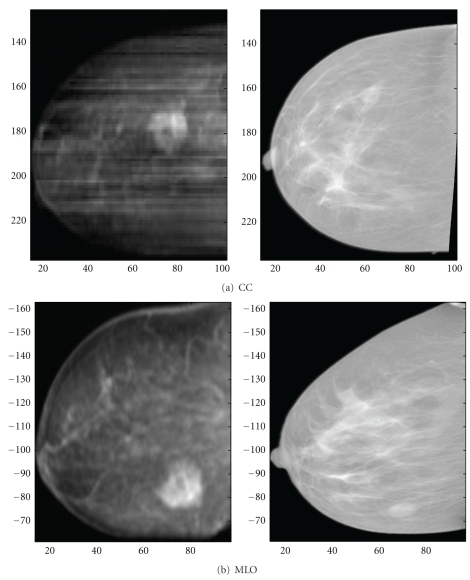
Comparison between contrast-enhanced MRPIs and registered mammograms of data set 100704-R. Corresponding lesions were indicated by a radiologist and are located to the right of centre in the CC images and on the lower right in the MLO images.

Figure 10 shows the locations of the lesion as identified by a radiologist and seen in the registered mammograms and corresponding MRPIs. The centroid of the CC lesion is located at approximately (75,180) in the MRPI and (65,165) in the mammogram, while the MLO lesion is at (75,85) and (65,70), respectively. This corresponds to less than an estimated 20° error in the “o'” clock frame of reference, which is well within the targeted “one hour” or 30° accuracy. In both the CC and the MLO views, the error was greatest in the lateral (*y* on a 2D image) direction. This is an expected result, as the greatest deformation is also expected to occur in this direction as can be observed in Figure 4.

In addition to the corresponding lesions, the radiologist identified other similarities indicative of accurate registration. While the CC projection image (Figure 10(a), left) is of low resolution and quality, the dark fatty regions appear to correspond with the dark fatty regions of the mammogram. On the MLO view, the external shape is less accurate due to the bulge and slight flattening of the MRPI; however, the internal glandular structures radiating from the nipple show good agreement. Since the subtraction image was not used for MI maximization, these features were not responsible for driving the registration process, illustrating the effectiveness of MI maximization in cases where corresponding features are not distinctly visible.

**Figure 11:**
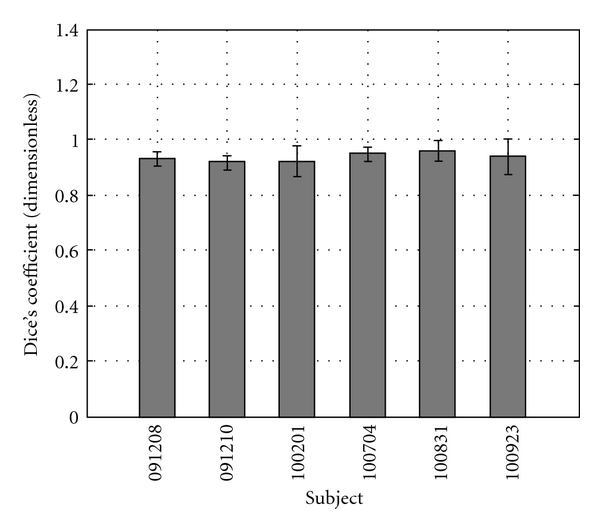
Average and standard deviation of Dice's coefficient for each data set of four image pairs.

**Figure 12:**
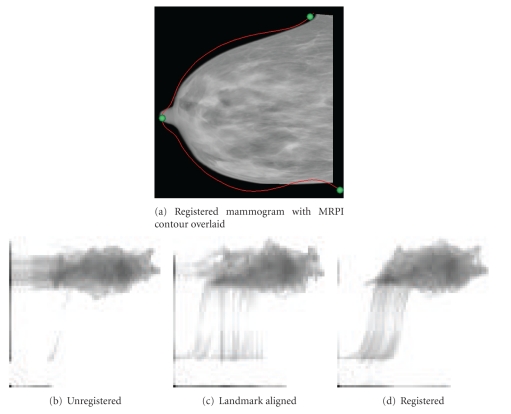
Registration results for sample MLO data set of Figures 4 and 5. The change in joint entropy plots can be observed as registration is achieved.

**Figure 13:**
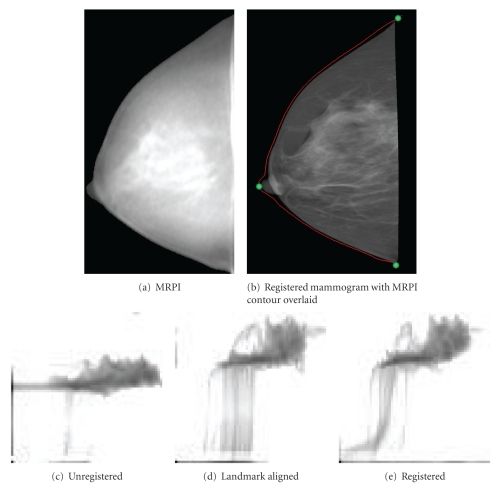
The data set with the highest Dice's coefficient value and most coherent entropy plot, objectively the best alignment (100923-R-MLO).

**Figure 14:**
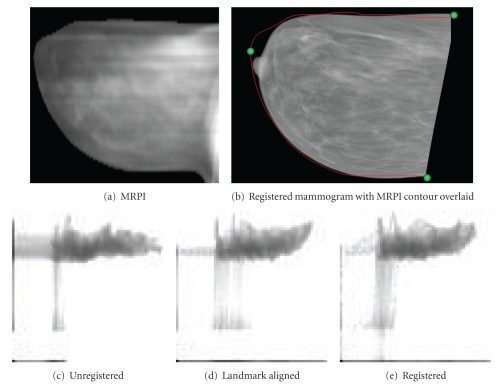
An example of deformation resulting from contact with the MR system (091210-L-CC).

The average of the Dice's coefficients for the four images in each data set is presented in Figure 11, with sample images shown in Figures 12–14. It can be seen that all of the images resulted in similar values averaging over 0.9 for Dice's coefficient. This means that the alignment of the external shape of each image pair was excellent, despite the high variability in breast shapes that can be observed by comparing Figures 12–14.

The joint entropy plots for the clinical images were computed with *N* = 64 histogram bins. Due to the large degree of complexity inherent to clinical images, relatively low resolution histograms were chosen to allow for clear changes to be observed.

Figure 12 shows the final registered mammogram from data set 091208-R-MLO, used to illustrate intermediate processing steps in Figures 4 and 5. The corresponding joint entropy plot following registration shows improved cohesiveness relative to the joint entropy plot prior to registration. While the joint entropy plots of patient data do not display the same degree of cohesiveness as the simulated data of Section 3, a clear trend of increasing coherence can be observed in Figures 12(b)–12(d). This indicates that MI maximization was successful without getting trapped in local maxima.

Figure 13 shows the image pair achieving the best correspondence. Dice's coefficient is the highest of all data sets at 0.99, and the final entropy plot is seen to have excellent coherence, indicating that local maxima were avoided. While quantification of internal feature registration is difficult, expert inspection of the two images concluded that the fat/glandular interface appears to be accurately located on the two images.

Figure 14 shows an example of a challenging registration case. In this data set, the breast was in contact with the MR coil, resulting in a distorted shape. Despite this confounding factor, the joint entropy plot is comparable to that of Figure 12(b). With the upturned nipple present in both cases, as well as good agreement between the fat/glandular interfaces seen most distinctly along the curvature of the breast below the nipple, expert inspection agreed that satisfactory registration was achieved even in this nonideal situation. Of the three data sets presented in Figures 12–14, large differences in breast shape can be observed. While registration accuracy is also variable, even the most challenging case shown in Figure 14 has achieved good correspondence between the two disparate modalities.

The registration algorithm was found to be efficient, with preprocessing requiring one minute per image, and registration taking an average of two minutes on a 2.4 GHz Intel Q6600 desktop processor.

## 5. Discussion

The aim of this work was to obtain an estimate of mammogram features in an undistorted 2D projection space, with the future goal of estimating the location of these features in 3D space to assist with TSAR imaging. To this end, an intensity-based method to map mammograms into the space of an undistorted 3D modality via 2D registration has been successfully demonstrated on six sets of patient images acquired using varying parameters. While MR images were used for the results presented in this paper, the algorithm could be used to register mammograms to other 3D modalities, such as PET or microwave images. Furthermore, this technique does not depend on the presence of corresponding internal landmarks or complicated models, making it fast and effective in any individual case. This emphasis on flexibility, automation, and efficiency opens up the possibility of using the technique in a clinical setting.

Just as it is difficult to assess the accuracy of a deformable registration method, objective comparison between this work and the three previous studies is difficult, as all have different goals and reporting methods. The method developed by Ruiter et al. was found to produce the most accurate results; similarly, the technique developed by Mertzanidou shows promise as a highly accurate registration technique [9, 10]. However, as the current work aims to map mammographic features into the space of the undistorted MRPI, results are not comparable to these techniques that find the opposite transformation.

Of the techniques most similar to this work, both Martí and Behrenbruch relied on identification of corresponding internal landmarks in order to achieve correspondence [7, 8]. In contrast, the methods introduced in this paper do not require such landmarks, eliminating any reliance on discrete features. In addition to requiring internal landmarks, Behrenbruch's work was limited by the use of corresponding lesions as salient landmarks for registration. This decreased the accuracy of data sets with larger pathologies, as well as those with features visible in only one modality, such as microcalcifications [7]. A key feature of the current work is the ability to achieve registration without clearly visible pathologies on both modalities.

To account for differences in resolution in the MLO and CC projection images, Behrenbruch reported lesion centroid errors relative to voxel size, with an average of 3–4.5 voxels depending on lesion type [7]. The same scaling applied to the current work results in a lesion centroid error of 2.7–16.8 voxels depending on view. While this seems significantly worse, the MR images of the current work are over three times the resolution; thus, the absolute (mm) errors represent a similar level of accuracy for the MLO view and about 30% worse for the CC view. However, Behrenbruch's work used lesions as landmarks, potentially resulting in locally accurate results without true registration. It is a promising result for the current work that comparable registration accuracy has been achieved despite the lesion only being visible on one modality.

With respect to the goal of obtaining “same hour” accuracy in locating features, it is expected that the current work is sufficiently accurate. This was demonstrated through the use of the numerical phantom of Section 3, where a maximum Euclidean distance of 2 mm error between lesion centroids was obtained. For the distal lesion, this corresponds to approximately 3°, much less than the 30° range of “one hour.” For the patient data set containing a lesion visible in the MR subtraction image, the error was estimated to be under 20°. While this is still within the “one hour” target, it is a greater error than found in the numerical phantom case. This can be attributed to the visibility of the lesion on both registered images in the phantom case; in effect, the lesion localization was partially responsible for driving the registration.

Several limitations of the current work can be readily identified. Most significantly, only a single data set containing verifiable internal feature locations was available. This limited the assessment of results to the metrics of Dice's coefficient and expert opinion; although these have been shown to have relevance in assessing accuracy, they are not as precise as true error computations. In addition, a larger number of data sets is required to truly assess robustness.

The works of Ruiter and Mertzanidou show that a 3D deformation model is more effective at representing the distortion resulting from mammographic compression [9, 10]. This is to be expected, as the physical process is indeed a 3D deformation. However, the goal of the current work was to map from the mammographic space to an “undistorted” space for future 3D reconstruction of mammographic features and comparison to TSAR images; thus, a 2D deformation model was chosen. This is an inherent limitation of the mapping direction chosen for this work and a source of potential inaccuracies.

Several further inaccuracies arise from uncertainties in the images themselves. Most notably, the true projection angles of the mammograms are subject to patient position variability. While the numerical phantom showed that the registration technique was relatively insensitive to small rotations in projection angle, the absolute accuracy of the registration is likely affected. Similarly, the amount of tissue imaged was difficult to match in cases where the chest wall was not visible on the mammogram; in these cases, the chest wall landmarks were taken to be on the edge of the image itself.

Another limitation is the manual intervention required to adjust parameters on a case-by-case basis. While the goal was to develop a fully automated registration technique, several parameters required modification to achieve convergence and avoid local minima. Fortunately, identification of these cases was obvious; registration tended to fail drastically or succeed.

## 6. Conclusion

A method for semiautomatically registering mammograms to projection images formed from MR image volumes has been developed and successfully tested on 24 image pairs from six patients. This demonstrates robustness of the algorithm to different breast shapes, imaging parameters, and tissue distributions. Subjective evaluation of images suggests that good registration was achieved, which is supported by high values of Dice's coefficient and increased coherency in joint entropy plots following registration.

Validation through the use of a numerical phantom further demonstrates the effectiveness of this registration method and increases confidence in the results. A high degree of accuracy in lesion localization on the registered phantom data was observed and found to be well within range of the targeted accuracy. Similarly, lesion localization on the single data set containing corresponding lesions was found to be within the “one hour” target and comparable to previous 2D registration techniques.

With an execution time in on the order of 1-2 minutes on relatively low-end hardware, the method is computationally efficient, while the use of a graphical interface allows for parametric refinements on the fly. These features make the technique user friendly and eliminate interoperator variability, allowing for possible clinical application.

Future work will be to test and refine the algorithm on an expanded data set, including more subjects with lesions visible on both modalities as well as TSAR data. In addition, a 3D model of the breast based on the undistorted mammograms will be created and used to assist with TSAR image interpretation.
